# Medically Assisted Reproduction and Risk of Cancer Among Offspring

**DOI:** 10.1001/jamanetworkopen.2024.9429

**Published:** 2024-05-02

**Authors:** Paula Rios, Philippe Herlemont, Patricia Fauque, Brigitte Lacour, Pierre Jouannet, Alain Weill, Mahmoud Zureik, Jacqueline Clavel, Rosemary Dray-Spira

**Affiliations:** 1 EPI-PHARE Scientific Interest Group in Epidemiology of Health Products, French National Agency for Medicines and Health Products Safety, French National Health Insurance, Saint-Denis, France; 2 Epidemiology of Childhood and Adolescent Cancers, Centre for Research in Epidemiology and Statistics, French National Institute for Health and Medical Research (INSERM) Joint Research Unit (UMR) 1153, Université Paris-Cité, Paris, France; 3 INSERM UMR 1231, Université Bourgogne Franche-Comté, Dijon, France; 4 French National Registry of Childhood Cancers, Assistance Publique–Hôpitaux de Paris, Centre Hospitalier Régional Universitaire (CHU) Paul Brousse, Villejuif, France; 5 French National Registry of Childhood Solid Tumours, CHU de Nancy, Nancy, France; 6 Université Paris-Cité, Paris, France

## Abstract

**Importance:**

Cancer is a leading cause of death among children worldwide. Treatments used for medically assisted reproduction (MAR) are suspected risk factors because of their potential for epigenetic disturbance and associated congenital malformations.

**Objective:**

To assess the risk of cancer, overall and by cancer type, among children born after MAR compared with children conceived naturally.

**Design, Setting, and Participants:**

For this cohort study, the French National Mother-Child Register (EPI-MERES) was searched for all live births that occurred in France between January 1, 2010, and December 31, 2021 (and followed up until June 30, 2022). The EPI-MERES was built from comprehensive data of the French National Health Data System. Data analysis was performed from December 1, 2021, to June 30, 2023.

**Exposure:**

Use of assisted reproduction technologies (ART), such as fresh embryo transfer (ET) or frozen ET (FET), and artificial insemination (AI).

**Main Outcomes and Measures:**

The risk of cancer was compared, overall and by cancer type, among children born after fresh ET, FET, or AI and children conceived naturally, using Cox proportional hazards regression models adjusted for maternal and child characteristics at birth.

**Results:**

This study included 8 526 306 children with a mean (SD) age of 6.4 (3.4) years; 51.2% were boys, 96.4% were singletons, 12.1% were small for gestational age at birth, and 3.1% had a congenital malformation. There were 260 236 children (3.1%) born after MAR, including 133 965 (1.6%) after fresh ET, 66 165 (0.8%) after FET, and 60 106 (0.7%) after AI. A total of 9256 case patients with cancer were identified over a median follow-up of 6.7 (IQR, 3.7-9.6) years; 165, 57, and 70 were born after fresh ET, FET, and AI, respectively. The overall risk of cancer did not differ between children conceived naturally and those born after fresh ET (hazard ratio [HR], 1.12 [95% CI, 0.96 to 1.31]), FET (HR, 1.02 [95% CI, 0.78 to 1.32]), or AI (HR, 1.09 [95% CI, 0.86 to 1.38]). However, the risk of acute lymphoblastic leukemia was higher among children born after FET (20 case patients; HR 1.61 [95% CI, 1.04 to 2.50]; risk difference [RD], 23.2 [95% CI, 1.5 to 57.0] per million person-years) compared with children conceived naturally. Moreover, among children born between 2010 and 2015, the risk of leukemia was higher among children born after fresh ET (45 case patients; HR, 1.42 [95% CI, 1.06 to 1.92]; adjusted RD, 19.7 [95% CI, 2.8 to 43.2] per million person-years).

**Conclusions and Relevance:**

The findings of this cohort study suggest that children born after FET or fresh ET had an increased risk of leukemia compared with children conceived naturally. This risk, although resulting in a limited number of cases, needs to be monitored in view of the continuous increase in the use of ART.

## Key Points

**Question:**

Does cancer risk differ among children born after fresh embryo transfer (ET), frozen embryo transfer (FET), or artificial insemination (AI) and children conceived naturally?

**Findings:**

In this cohort study of 8 526 306 children, the overall risk of cancer did not differ among children born after fresh ET, FET, or AI and children conceived naturally. However, an increased risk of leukemia was observed among children born after fresh ET (especially those with the longest follow-up) and among children born after FET (particularly acute lymphoblastic leukemia).

**Meaning:**

These findings suggest that the risk of leukemia among children conceived by FET or fresh ET should be further monitored.

## Teaser

This cohort study assesses the risk of cancer, overall and by cancer type, among children born after medically assisted reproduction compared with children conceived naturally.

## Introduction

Childhood cancer is a leading cause of death among children worldwide. Overall, 1800 cases occur every year in France among children aged younger than 15 years, with half occurring before age 6 years.^1^ The early onset after birth and the embryologic characteristics of some cancers^2^ suggest that the causes may take place around conception. Treatments involved in medically assisted reproduction (MAR),^3^ including assisted reproduction technologies (ART; eg, fresh embryo transfer [ET] or frozen ET [FET]) and artificial insemination (AI), have been suggested as potential risk factors for childhood cancer.^4,5,6^

Children born after MAR represent a growing population worldwide.^7^ Compared with children conceived naturally, children born after MAR have an increased risk of adverse perinatal outcomes, including preterm birth, low birth weight, and fetal growth anomalies.^8,9^ However, little is known about the medium-term and long-term effects of fertility treatments on child health. Previous studies on the risk of childhood cancer among children born after ART, most of which were based on relatively small cohorts of exposed children^10,11,12,13,14,15,16,17,18^ or interview-based case-control study designs,^19,20,21,22,23,24^ provided contrasting results. With regard to findings from large cohorts including 100 000 or more exposed children,^25,26,27,28,29^ no increase in overall cancer risk has been reported, except 1 study^29^ that reported a marginal association with ART. Nevertheless, positive associations have been reported when investigating specific cancer types.^25,26,27,28,29^ In addition, previous studies that assessed the risk of childhood cancer by fresh ET or FET^12,15,17,25,26,27,28^ were based on limited numbers of case patients exposed to each ART modality.

We aimed to assess the risk of cancer, overall and by cancer type, among children born after ART use (fresh ET or FET) or after AI compared with children conceived naturally, considering all children born in France between 2010 and 2021.

## Methods

This cohort study was authorized by decree 2016-1871 from December 26, 2016, relating to the processing of personal data from the National Health Data System and French law Art. 1461-13 and 14. The EPI-PHARE Scientific Interest Group in Epidemiology of Health Products, which has permanent regulatory access to the data from the French National Health Data System (SNDS; in application of the provisions of Art. R. 1461-12 et seq. 9 of the French Public Health Code and French Data Protection Authority decision CNIL-2016-10 316), was exempted from participant consent and institutional review board approval. The study was registered in the EPI-PHARE SNDS-based registry under reference T-2020-10-272. The study followed the Strengthening the Reporting of Observational Studies in Epidemiology (STROBE) reporting guideline.

### Data Source

This study was conducted using comprehensive data from the French National Mother-Child Register (EPI-MERES) for all children born alive in France between January 1, 2010, and December 31, 2021. The EPI-MERES database was built from the SNDS,^30^ which provides comprehensive hospitalization and health insurance reimbursement data for more than 99% of the French population; it is an unselected register of all pregnancies from 2010 onward in France, identified by end-of-pregnancy diagnoses and procedure codes and linked to their offspring using a deterministic algorithm. Linkage of mother and infant records is available for 95% of children. As of today, the EPI-MERES includes data for more than 8.5 million liveborn children, accounting for 98% of all live births recorded in France since 2010. The EPI-MERES contains a wide set of information on mothers (medical history and sociodemographic characteristics), pregnancies (start and end dates, mode of delivery, and pregnancy outcome), and offspring (gestational age and birth weight, outpatient and inpatient health care use, and hospital discharge diagnoses and medical procedures coded according to the *Classification Commune des Actes Médicaux* [*CCAM*]). Patients with long-term diseases (LTDs), such as cancer, benefit from full coverage of their health expenditure, and the diagnosis is recorded in the Datamart Consommation Inter Régime (DCIR) using *International Statistical Classification of Diseases, Tenth Revision* (*ICD-10*) codes. The DCIR is a French health care expenditure database that contains all of the individual data of health insurance beneficiaries. Data from the EPI-MERES have been used previously to conduct drug use and safety pharmacoepidemiologic studies.^31,32,33,34^

### Exposure Assessment

Artificial insemination and ART are fully covered by French health insurance after medical validation and until a maternal age of 43 years. Procedures related to MAR and their dates were identified using *CCAM* codes for oocyte retrieval, ET, and AI (eTable 1 in Supplement 1). Children were considered as born after MAR when the conception date fell within 21 days of the MAR procedure recorded in the SNDS, and as naturally conceived otherwise. The recorded date of ET was retained as the ART procedure date. Children were considered as born after fresh ET when there were fewer than 21 days between the date of oocyte retrieval and the ET, and as born after FET when the delay was 21 days or longer.

### Childhood Cancer

Childhood cancers were identified using hospital discharge and LTD diagnoses, using algorithms constructed in collaboration with experts in cancer coding from the French National Registry of Childhood Cancers (RNCE). Children were considered case patients with cancer if they met at least 1 of the following criteria: (1) had at least 2 hospitalizations with a discharge diagnosis of cancer; (2) had at least 1 hospitalization with a discharge diagnosis of cancer in addition to 1 hospitalization for oncologic treatment (chemotherapy, radiotherapy, oncologic surgery, or bone marrow transplantation) or an LTD diagnosis of childhood cancer; or (3) had 1 hospitalization with a discharge diagnosis of cancer, followed by death within 6 months.

Cancer diagnoses were categorized into 12 groups as close as possible to those of the *International Classification of Childhood Cancer, Third Edition* (*ICCC-3*; eTable 2 in Supplement 1),^35^ using *ICD-10* codes, in the absence of the morphology and topography codes of the *International Classification of Diseases for Oncology, Third Edition* (*ICD-O-3*) required for *ICCC-3*. For each child, the most frequently coded cancer diagnostic group among all their hospital stays was retained as the cancer diagnosis. Because of a lack of specificity, for malignant bone tumors and gonadic neoplasms, only case patients with identification of an oncologic treatment were retained. Because information available in the SNDS does not allow for the proper individual identification of same-sex siblings born of multiple pregnancies, only 1 case patient was retained in the case of identical cancer diagnosis in same-sex twins. Anonymized data provided by the RNCE for children born and diagnosed in the 2010 to 2015 period were used as the reference set for the distribution in large categories of cancer diagnoses (eTable 3 in Supplement 1).

### Covariates

Characteristics of children included year of birth, sex, singleton or multiple birth, birth weight (<1000, 1000-2499, 2500-3999, or ≥4000 g), gestational length (≤28, 29-32, 33-36, 37-41, or ≥42 weeks), and an indicator of fetal growth (small, adequate, or large for gestational age). Major congenital malformations were identified based on hospital discharge diagnoses within up to 12 months after birth, based on *ICD-10* codes selected by the European Surveillance of Congenital Anomalies network.^36^ Characteristics of mothers included maternal age at the index child birth (≤24, 25-29, 30-34, 35-39, or ≥40 years) and the French census-based deprivation index^37^ of the municipality of residence at birth, used as a proxy for the household socioeconomic category. The French census-based deprivation index is categorized in quintiles, with the fifth quintile representing the most deprived municipalities.

### Statistical Analysis

Children contributed to person-years at risk from their date of birth until the date of first hospitalization with a discharge diagnosis of cancer, the date of death, or the end of the study on June 30, 2022, whichever occurred first. We compared the risk of cancer among children born after fresh ET, FET, or AI with that of children conceived naturally, taking all cancers together and by main cancer groups. The risk of leukemia was examined overall and by leukemia subtype (acute lymphoblastic leukemia [ALL] and acute myeloid leukemia [AML]).

Adjusted hazard ratios (HRs) and 95% CIs were estimated using Cox proportional hazards regression models including a priori selected covariates reported to be associated with childhood cancers and MAR in the literature (ie, sex, year of birth, multiple birth, maternal age at childbirth, and deprivation index). Additional models also adjusted for potential mediating factors including gestational length, birth weight, macrosomia, and congenital malformations. The percentages of missing data were small. To assess cancer risk among children followed over a longer period, complementary analyses restricted to children born between 2010 and 2015 were performed for most frequent childhood cancers. Partial likelihood tests were performed post hoc to determine whether the estimated HR differed across the various MAR modalities. Sensitivity analyses restricted to singletons were performed. Additional sensitivity analyses were performed using the first coded (instead of the most frequently coded) diagnosis group as the cancer diagnosis and restricting the case definition to children with chemotherapy exposure. The proportional hazards assumption was tested with Schoenfeld residuals, and there were no clear violations. Hazard ratios were not estimated for categories with fewer than 5 case patients. All *P* values were 2-sided and a significance level of .05 was applied. Data analysis was performed from December 1, 2021, to June 30, 2023, using SAS, version 9.4 (SAS Institute Inc).

## Results

### Characteristics of the Study Population

The study cohort included 8 526 306 children, with a mean (SD) age of 6.4 (3.4) years. Boys comprised 51.2% of the cohort, and girls comprised 48.8%; 96.4% of children were singletons, 12.1% were small for gestational age at birth, and 3.1% had a congenital malformation. There were 260 236 children (3.1%) born after MAR, including 133 965 (1.6%) after fresh ET, 66 165 (0.8%) after FET, and 60 106 (0.7%) after AI. Although the number of births after fresh ET decreased from 2015 onward, a continuous increase in the number of children born after FET was observed over the study period and FET started to prevail over fresh ET in 2020. The annual number of children born after AI remained stable over the study period (Figure).

**Figure.**
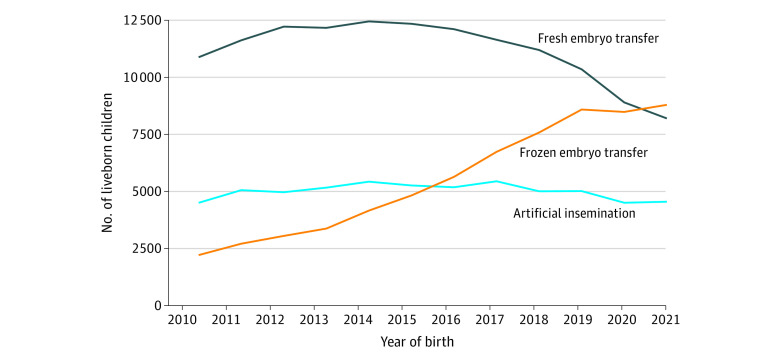
Children Born in France Between 2010 and 2021 After Medically Assisted Reproduction

Compared with children conceived naturally, children born after MAR were more often part of a multiple birth, had lower birth weight and gestational age, were more often born small for gestational age, and were more often diagnosed with a congenital malformation. Children born after FET were more frequently born large for gestational age. Mothers whose children were born after MAR were older and their residence at birth was more often in the least deprived municipalities (Table 1).

**Table 1.** Perinatal Characteristics of Children Born in France Between 2010 and 2021, According to Mode of Conception^a^

Characteristic	Children conceived naturally (n = 8 266 070)	Children born after MAR		
		Fresh ET (n = 133 965)	FET (n = 66 165)	AI (n = 60 106)
Child sex				
Male	4 229 103 (51.2)	67 669 (50.5)	33 204 (50.2)	30 871 (51.4)
Female	4 036 408 (48.8)	66 234 (49.4)	32 935 (49.8)	29 215 (48.6)
Missing	559 (0.01)	62 (0.05)	26 (0.04)	20 (0.03)
Multiplicity				
No	8 013 531 (96.9)	101 862 (76.0)	56 177 (84.9)	48 364 (80.5)
Yes	252 539 (3.1)	32 103 (24.0)	9988 (15.1)	11 742 (19.5)
Gestational length, wk				
≤28	23 249 (0.3)	1160 (0.9)	515 (0.8)	418 (0.7)
29-32	53 342 (0.6)	3023 (2.3)	1005 (1.5)	1021 (1.7)
33-36	479 004 (5.8)	21 280 (15.9)	7852 (11.9)	7984 (13.3)
37-41	7 640 605 (92.4)	107 883 (80.5)	56 110 (84.8)	50 381 (83.8)
≥42	69 870 (0.8)	619 (0.5)	683 (1.0)	302 (0.5)
Birth weight, g				
Missing	250 679 (3.0)	4091 (3.1)	2046 (3.1)	1925 (3.2)
<1000	25 872 (0.3)	1311 (1.0)	464 (0.7)	473 (0.8)
1000-2499	533 035 (6.4)	26 408 (19.7)	8308 (12.6)	9602 (16.0)
2500-3999	6 904 377 (83.5)	97 464 (72.8)	50 653 (76.6)	45 375 (75.5)
≥4000	552 107 (6.7)	4691 (3.5)	4694 (7.1)	2731 (4.5)
Birth weight, median (IQR), g	3290 (2970-3600)	3030 (2585-3410)	3230 (2820-3590)	3120 (2700-3480)
Fetal growth				
Missing	250 681 (3.0)	4091 (3.1)	2046 (3.1)	1925 (3.2)
Small for gestational age	977 847 (11.8)	27 995 (20.9)	8875 (13.4)	10 863 (18.1)
Adequate for gestational age	6 154 480 (74.5)	92 483 (69.0)	47 536 (71.8)	42 271 (70.3)
Large for gestational age	883 062 (10.7)	9396 (7.0)	7708 (11.7)	5047 (8.4)
Congenital malformations				
No	8 013 422 (96.9)	128 276 (95.8)	63 456 (95.9)	57 873 (96.3)
Yes	252 647 (3.1)	5689 (4.2)	2709 (4.1)	2233 (3.7)
Maternal age at index child birth, y				
≤24	1 638 340 (19.8)	4782 (3.6)	1830 (2.8)	2731 (4.5)
25-29	2 175 961 (26.3)	22 436 (16.7)	9633 (14.6)	12 143 (20.2)
30-34	2 732 138 (33.0)	54 157 (40.4)	27 046 (40.9)	24 800 (41.3)
35-39	1 371 193 (16.6)	42 384 (31.6)	21 700 (32.8)	16 464 (27.4)
≥40	348 438 (4.2)	10 206 (7.6)	5956 (9.0)	3968 (6.6)
Maternal age, median (IQR), y	30 (26-34)	33 (30-36)	34 (31-37)	33 (30-36)
Deprivation index				
Missing	121 499 (1.5)	2119 (1.6)	1091 (1.6)	1041 (1.7)
Overseas departments and regions	346 617 (4.2)	2862 (2.1)	1602 (2.4)	622 (1.0)
Q1 (least deprived)	1 560 924 (18.9)	32 391 (24.2)	16 771 (25.3)	15 958 (26.5)
Q2	1 569 456 (19.0)	27 965 (20.9)	13 999 (21.2)	13 209 (22.0)
Q3	1 543 164 (18.7)	25 146 (18.8)	12 214 (18.5)	10 832 (18.0)
Q4	1 506 618 (18.2)	22 829 (17.0)	10 658 (16.1)	9975 (16.6)
Q5 (most deprived)	1 617 792 (19.6)	20 653 (15.4)	9830 (14.9)	8469 (14.1)

Abbreviations: AI, artificial insemination; ET, embryo transfer; FET, frozen embryo transfer; MAR, medically assisted reproduction; Q, quintile.

^a^ Unless indicated otherwise, values are expressed as No. (%) of children.

### Cancer Risk Among Children Born After Fresh ET, FET, or AI

During a median follow-up of 6.7 (IQR, 3.7-9.6) years for children conceived naturally and 6.8 (IQR, 3.4-9.1) years, 4.4 (IQR, 2.4-7.3) years, and 6.5 (IQR, 3.6-9.4) years for children born after fresh ET, FET, and AI, respectively, 9256 case patients with cancer were identified. A total of 8964 cases were observed among children conceived naturally (incidence rate [IR], 164 per million person-years), 165 among children born after fresh ET (IR, 183 per million person-years), 57 among children born after FET (IR, 172 per million person-years), and 70 among children born after AI (IR, 179 per million person-years). Case patients with cancer more often were born preterm, were large for gestational age, and were diagnosed with congenital malformation compared with children without cancer (eTable 4 in Supplement 1). The overall risk of cancer did not differ between children conceived naturally and those born after fresh ET (HR, 1.12 [95% CI, 0.96 to 1.31]), FET (HR, 1.02 [95% CI, 0.78 to 1.32]), or AI (HR, 1.09 [95% CI, 0.86 to 1.38]) (Table 2).

**Table 2.** Risk of Cancer Among Children Born in France Between 2010 and 2021 After Fresh ET, FET, or AI Compared With Children Conceived Naturally^a^

Childhood cancer type	No. (IR) of children conceived naturally (n = 8 266 070)	Children born after MAR^b^								
		Fresh ET (n = 133 965)			FET (n = 66 165)			AI (n = 60 106)		
		No. (IR) of case patients	HR (95% CI)	RD (95% CI)	No. (IR) of case patients	HR (95% CI)	RD (95% CI)	No. (IR) of case patients	HR (95% CI)	RD (95% CI)
Any cancer	8964 (164)	165 (183)	1.12 (0.96 to 1.31)	19.7 (−6.6 to 50.8)	57 (172)	1.02 (0.78 to 1.32)	3.3 (−36.1 to 52.4)	70 (179)	1.09 (0.86 to 1.38)	14.7 (−22.9 to 62.3)
Leukemia	2635 (48)	52 (58)	1.19 (0.90 to 1.56)	9.1 (−4.8 to 26.9)	23 (69)	1.42 (0.94 to 2.14)	20.1 (−2.8 to 54.7)	19 (49)	1.01 (0.64 to 1.58)	0.8 (−17.2 to 27.8)
ALL	2 083 (38)	39 (43)	1.14 (0.83 to 1.57)	5.3 (−6.7 to 21.7)	20 (60)	1.61 (1.04 to 2.50)	23.2 (1.5 to 57.0)	16 (41)	1.09 (0.66 to 1.78)	3.4 (−12.9 to 29.6)
AML	475 (9)	10 (11)	1.20 (0.64 to 2.25)	1.8 (−3.2 to 11.2)	<5	NA	NA	<5	NA	NA
Lymphoma	801 (15)	13 (14)	1.10 (0.63 to 1.90)	1.5 (−5.5 to 13.5)	<5	NA	NA	6 (15)	1.13 (0.50 to 2.52)	1.9 (−7.5 to 22.8)
Malignant CNS tumor	1813 (33)	35 (39)	1.23 (0.87 to 1.72)	7.5 (−4.3 to 23.7)	8 (24)	0.78 (0.39 to 1.59)	−7.2 (−20.1 to 19.4)	19 (48)	1.51 (0.92 to 2.38)	16.8 (−2.6 to 45.5)
Embryonal tumor	2406 (44)	41 (45)	1.05 (0.77 to 1.43)	2.2 (−10.1 to 18.9)	16 (45)	0.96 (0.59 to 1.57)	−1.7 (−18.0 to 25.0)	18 (46)	1.05 (0.66 to 1.67)	2.2 (−14.9 to 29.4)
Adrenal gland tumor^c^	941 (17)	20 (22)	1.35 (0.86 to 2.10)	5.9 (−2.3 to 18.7)	6 (18)	0.94 (0.42 to 2.09)	−1.0 (−9.8 to 18.5)	9 (23)	1.38 (0.72 to 2.77)	6.4 (−4.7 to 30.1)
Retinoblastoma	507 (9)	10 (11)	1.18 (0.63 to 2.23)	1.6 (−3.3 to 11.1)	<5	NA	NA	<5	NA	NA
Renal tumor	777 (14)	10 (11)	0.81 (0.43 to 1.51)	−2.6 (−7.9 to 7.1)	7 (21)	1.36(0.64 to 2.85)	5.0 (−5.0 to 25.9)	6 (15)	1.09 (0.49 to 2.45)	1.2 (−7.1 to 20.3)
Hepatic tumor	181 (3)	<5	NA	NA	0	NA	NA	<5	NA	NA
Malignant bone tumor	235 (4)	<5	NA	NA	0	NA	NA	<5	NA	NA
Soft tissue sarcoma	519 (10)	11 (12)	1.29 (0.70 to 2.35)	2.9 (−3.0 to 13.5)	<5	NA	NA	<5	NA	NA
Germ cell and gonad tumor	117 (2)	<5	NA	NA	<5	NA	NA	0	NA	NA
Epithelial neoplasm and melanoma	306 (6)	8 (9)	1.32 (0.65 to 2.68)	1.9 (−2.1 to 10.1)	<5	NA	NA	<5	NA	NA
Other and unspecified neoplasm	132 (2)	<5	NA	NA	0	NA	NA	<5	NA	NA

Abbreviations: AI, artificial insemination; ALL, acute lymphoblastic leukemia; AML, acute myeloid leukemia; CNS, central nervous system; ET, embryo transfer; FET, frozen embryo transfer; HR, hazard ratio; IR, incidence rate per million person-years; MAR, medically assisted reproduction; NA, not applicable; RD, risk difference per million person-years.

^a^ Estimates are not provided when there were fewer than 5 exposed case patients. The number of person-years was 54 568 974 for children conceived naturally and 903 708, 331 770, and 391 001 for children conceived via fresh ET, FET, and AI, respectively.

^b^ All HR and RD values are adjusted for year of birth, sex, multiple birth, maternal age, and deprivation index.

^c^ Includes tumors of adrenal glands, peripheral nerves, and autonomic nervous system.

Leukemia was the most commonly diagnosed type of cancer, accounting for 29.4% of total cases. Among these case patients, 2635 were children conceived naturally (IR, 48 per million person-years), 52 were born after fresh ET (IR, 58 per million person-years), 23 were born after FET (IR, 69 per million person-years), and 19 were born after AI (IR, 49 per million person-years) (Table 2). Acute lymphoblastic leukemia accounted for 79.1% of leukemia cases. Among these case patients, 2 083 were children conceived naturally (IR, 38 per million person-years), 39 were born after fresh ET (IR, 43 per million person-years), 20 were born after FET (IR, 60 per million person-years), and 16 were born after AI (IR, 41 per million person-years). Overall, the risk of leukemia for children born after MAR did not differ significantly from that of children conceived naturally (fresh ET: HR, 1.19 [95% CI, 0.90 to 1.56]; FET: HR, 1.42 [95% CI, 0.94 to 2.14; and AI: HR, 1.01 [95% CI, 0.64 to 1.58]). The risk of ALL was significantly increased after FET (HR, 1.61 [95% CI, 1.04 to 2.50]; adjusted risk difference [RD], 23.2 [95% CI, 1.5 to 57.0] per million person-years), but not after fresh ET (HR, 1.14 [95% CI, 0.83 to 1.57]) (Table 2). The partial likelihood test was not statistically significant (*P* = .07).

Besides leukemia, the most frequent groups of cancers were tumors of the central nervous system (CNS; n = 1875), embryonal tumors (n = 2481), and lymphomas (n = 823). Compared with children conceived naturally, the risk of each of these cancer groups did not differ among children born after fresh ET or among those born after FET. The risk of malignant CNS tumors tended to be higher among children born after AI, although estimates did not reach statistical significance (HR, 1.51 [95% CI, 0.92 to 2.38]; adjusted RD, 16.8 [95% CI, –2.6 to 45.5] per million person-years) (Table 2).

In the analyses restricted to children born between 2010 and 2015, the median follow-up duration was longer and more homogeneous for the different modes of conception. These median durations were as follows: 9.4 (IQR, 7.8-10.9) years for natural conception, 9.4 (IQR, 7.9-10.9) years for fresh ET, 8.8 (IQR, 7.5-10.4) years for FET, and 9.3 (IQR, 7.9-10.8) years for AI. The risk of leukemia was significantly increased among children born after fresh ET (HR, 1.42 [95% CI, 1.06 to 1.92]; adjusted RD, 19.7 [95% CI, 2.8 to 43.2] per million person-years) but not after FET (HR, 1.27 [95% CI, 0.70 to 2.29]). The partial likelihood test was not statistically significant (*P* = .06). The risk of ALL was increased, without reaching statistical significance, among children born after fresh ET (HR, 1.33 [95% CI, 0.93 to 1.89]; adjusted RD, 12.5 [95% CI, – 2.7 to 33.8] per million person-years) and FET (HR, 1.47 [95% CI, 0.79 to 2.74]; adjusted RD, 17.9 [95% CI, −8.0 to 66.1] per million person-years). No other association was observed (Table 3).

**Table 3.** Risk of Cancer Among Children Born in France Between 2010 and 2015 After Fresh ET, FET, and AI Compared With Children Conceived Naturally^a^

Childhood cancer type	No. (IR) of children conceived naturally (n = 4 279 407)	Children born after MAR^b^								
		Fresh ET (n = 71 617)			FET (n = 20 360)			AI (n = 30 391)		
		No. (IR) of case patients	HR (95% CI)	RD (95% CI)	No. (IR) of case patients	HR (95% CI)	RD (95% CI)	No. (IR) of case patients	HR (95% CI)	RD (95% CI)
Any cancer	6 336 (157)	125 (186)	1.18 (0.99 to 1.42)	28.3 (−1.6 to 65.9)	30 (163)	1.03 (0.72 to 1.48)	4.7 (−43.9 to 75.4)	54 (189)	1.20 (0.92 to 1.57)	31.4 (−12.6 to 89.5)
Leukemia	1 897 (47)	45 (67)	1.42 (1.06 to 1.92)	19.7 (2.8 to 43.2)	11 (60)	1.27 (0.70 to 2.29)	12.7 (−14.1 to 60.6)	16 (56)	1.19 (0.72 to 1.94)	8.9 (−13.2 to 44.2)
ALL	1 527 (38)	33 (49)	1.33 (0.93 to 1.89)	12.5 (−2.7 to 33.8)	10 (54)	1.47 (0.79 to 2.74)	17.9 (−8.0 to 66.1)	14 (49)	1.32 (0.78 to 2.24)	12.2 (−8.4 to 47.2)
AML	313 (7)	9 (13)	1.54 (0.79 to 3.01)	3.8 (−1.5 to 14.1)	<5	NA	NA	<5	NA	NA
Lymphoma	648 (16)	9 (14)	0.94 (0.48 to 1.82)	−1 (−8.3 to 13.1)	<5	NA	NA	5 (17)	1.17 (0.49 to 2.84)	2.7 (−8.2 to 29.4)
Malignant CNS tumor	1 355 (33)	26 (39)	1.20 (0.82 to 1.79)	6.6 (−5.9 to 26.1)	6 (33)	1.02 (0.46 to 2.28)	0.6 (−17.8 to 42.2)	15 (53)	1.60 (0.96 to 2.68)	19.8 (−1.3 to 55.4)
Embryonal tumor^c^	1378 (34)	24 (35)	1.05 (0.70 to 1.58)	1.7 (−10.2 to 19.7)	8 (43)	1.25 (0.62 to 2.51)	8.5 (−12.9 to 51.3)	14 (49)	1.45 (0.86 to 2.45)	15.3 (−4.8 to 49.3)

Abbreviations: AI, artificial insemination; ALL, acute lymphoblastic leukemia; AML, acute myeloid leukemia; CNS, central nervous system; ET, embryo transfer; FET, frozen embryo transfer; HR, hazard ratio; IR, incidence rate per million person-years; MAR, medically assisted reproduction; NA, not applicable; RD, risk difference per million person-years.

^a^ Estimates are not provided when there were fewer than 5 exposed case patients. The number of person-years was 40 434 932 for children conceived naturally and 672 874, 183 818, and 284 868 for children conceived via fresh ET, FET, and AI, respectively.

^b^ Adjusted for year of birth, sex, multiple birth, maternal age, and deprivation index.

^c^ Includes tumors of adrenal glands, peripheral nerves, and autonomic nervous system.

Additional adjustments for birth characteristics and congenital malformations marginally modified the estimates (eTables 5-7 in Supplement 1). Results remained stable in sensitivity analyses restricted to singletons or based on alternative case definitions (eTables 8-10 in Supplement 1).

## Discussion

In this nationwide birth cohort study including more than 8.5 million children, the risk of any cancer was not increased among children born after MAR. However, the findings suggest that children born after fresh ET or FET had a higher risk of leukemia.

The absence of an overall association between cancer and MAR in this study is consistent with the findings of most large observational studies.^25,26,27,28^ However, the grouping of all cancer types, although meaningful for detecting common risk factors, could miss subtype specificities. In line with our findings, 4 previous studies^13,17,27,28^ reported an increased risk of leukemia among children born after ART. With regard to ART modalities such as fresh ET or FET, the literature is still sparse and results are conflicting. The data used in this study were obtained from one of the largest cohorts of children born after FET to date; thus, our results support the hypothesis of an increased risk of leukemia, mainly observable for ALL, with a limited number for AML. In our complementary analysis restricted to births between 2010 and 2015, the risk of leukemia was increased for children born after FET (although statistical significance was not reached because of reduced numbers of exposed case patients) and also for those born after fresh ET, probably as a result of more homogeneous follow-up between children born after fresh ET or FET and better coverage of the age range corresponding to the incidence peak of leukemia (2-6 years). Sargisian et al^28^ conducted a study (median follow-up of 9.9 years) in Nordic countries, and they found an increased risk of leukemia among children born after FET vs children conceived naturally (HR, 2.22 [95% CI, 1.47 to 3.35]) but not among those born after fresh ET. In another study, Weng et al^17^ found that the risk of leukemia was significantly increased among children born after fresh ET, but not among children born after FET. However, estimates were based on fewer than 5 exposed case patients by ART modality. A meta-analysis conducted by Zhang et al^38^ reported no increased cancer risk among children born after ART based on 15 cohorts, but the authors found an increased risk of childhood cancer among children born after FET (pooled HR, 1.37 [95% CI, 1.04 to 1.81]) compared with children conceived naturally. However, the finding was not statistically significant when children born after fresh ET were considered as the reference group (pooled HR, 1.28 [95% CI, 0.96 to 1.69]), indicating that the increased incidence of cancer among children born after FET may be based on in vitro fertilization (IVF), intracytoplasmic sperm injection (ICSI), or any other ART. Differences between studies might be explained by heterogeneity across them in terms of sample size, follow-up duration, and study period, because substantial changes in ART practices occurred over time within the same laboratory and among different laboratories (eg, culture conditions: culture media used and oxygen tension, or embryo freezing processes).

Our findings do not provide evidence of an increased risk of other types of childhood cancer among children born after MAR. Based on 19 exposed case patients, the nonsignificantly increased risk of CNS tumors after AI reported here warrants further investigation. Furthermore, the number of hepatic tumors among exposed children was very small (<5 after fresh ET and 0 after FET), and the overall risk of embryonal tumors was not increased among children born after MAR.

The etiologic mechanisms behind a possible increased risk of cancer among children born after MAR are not known, but explanatory hypotheses consider epigenetic disturbance as a potential pathway. Previous studies have found that procedures involved in MAR, such as IVF, ICSI, and ovarian stimulation, can induce epigenetic changes in human embryos, cord blood, and placentae.^39,40^ This finding is also supported by studies that suggest an association between MAR-related procedures and imprinting disorders,^41^ such as Beckwith-Weidemann syndrome,^42^ a pediatric overgrowth disorder involving a predisposition to tumor development.

### Strengths and Limitations

Our study has major strengths. It is based on one of the largest cohorts published to date and on comprehensive data covering the entire French population, and we used reliable and systematically collected data, with no potential for recall bias and selection bias. We performed analyses for the most frequent childhood cancer groups and by MAR modality, and we had reliable individual information on major confounders and potential mediating factors.

Our findings must be interpreted in light of some limitations. We used reimbursement and hospitalization data to identify exposure to MAR procedures and childhood cancer occurrence. With regard to exposure assessment, MAR-related procedures are expensive treatments that benefit from full insurance coverage in France; therefore, they typically are not subject to nondeclaration. Compared with reported numbers from the French Agency of Biomedicine over the same period, our study was able to identify more than 90% of liveborn children conceived by MAR, and the distribution by fresh ET, FET, or AI was comparable (eTable 11 in Supplement 1). Still, information on the type of ART (ie, ICSI) or cross-border fertility care was not available.

With regard to outcome assessment, most pediatric cancers are life-threatening pathologies requiring hospitalization for diagnosis and treatment and are very unlikely to be underdiagnosed in France. The algorithms used in our study were able to identify the most frequent groups of childhood cancers with numbers close to that of the RNCE except for tumors of the adrenal glands, peripheral nerves, and autonomic nervous system, which were underestimated in our study based on *ICD-10* diagnoses. Conversely, the number of bone tumors, hepatic tumors, and malignant CNS tumors was higher than that of the registry, possibly reflecting inclusions of metastatic localizations as primary sites. Exposure or outcome misclassification might have attenuated the associations.

Additionally, information on child emigration was not available. Given the rarity of childhood cancers, the number of potential undetected cases because of child emigration is likely to be small. Child emigration could also lead to overestimation of the follow-up time. However, these potential misclassification biases might not differ by conception mode; therefore, they should not have substantially biased our estimates.

Many potential confounders have been taken into account. However, as with most studies on the effects of fertility treatments on child health, our study was unable to distinguish between possible treatment effects and parental conditions underlying infertility^43^ that might be associated with an increased risk of cancer in offspring. Therefore, residual confounding cannot be completely ruled out.

Finally, although this study included a large nationwide cohort, the absolute number of children with cancer was relatively small. Risk assessment of childhood cancers is challenging given their rarity, and results based on small numbers may be subject to both lack of power or the potential for spurious associations. We did not adjust for multiple comparisons. Therefore, these findings must be interpreted with caution.

## Conclusions

In this cohort study, the overall risk of cancer was not increased after MAR but our findings suggest that children born after FET or fresh ET may have an increased risk of leukemia. This risk, although resulting in a limited number of cases, needs to be monitored in view of the continuous increase in the use of ART.

## Notes

Supplement 1. **eTable 1.** List of Codes Used for Identification of Medically Assisted Reproduction–Related Procedures **eTable 2.** Grouping of *ICD-10* Diagnostic Codes Used to Approximate the International Childhood Cancer Classification **eTable 3.** Number of Cancers Among Children Born and Diagnosed in France Between 2010 and 2015 in the French National Registry of Childhood Cancers (RNCE) and in the Present Study **eTable 4.** Birth Characteristics of Children Born in France in 2010 to 2021 by Cancer Status **eTable 5.** Risk of Cancer in Children Born in France in 2010 to 2021 After Fresh Embryo Transfer Compared to Naturally Conceived Children **eTable 6.** Risk of Cancer in Children Born in France in 2010 to 2021 After Frozen Embryo Transfer Compared to Naturally Conceived Children **eTable 7.** Risk of Cancer in Children Born in France in 2010 to 2021 After Artificial Insemination Compared to Naturally Conceived Children **eTable 8.** Number of Cases, Incidence Rate, and Risk of Cancer in Children Born After Natural Conception, Fresh Embryo Transfer, Frozen Embryo Transfer, or Artificial Insemination in Singletons Born in France in 2010 to 2021 **eTable 9.** Risk of Cancer in Children Born in France in 2010 to 2021 After Fresh Embryo Transfer, Frozen Embryo Transfer, or Artificial Insemination Compared to Naturally Conceived Children **eTable 10.** Risk of Cancer in Children Born in France in 2010 to 2021 After Fresh Embryo Transfer, Frozen Embryo Transfer, or Artificial Insemination Compared to Naturally Conceived Children **eTable 11.** Number of Children Born After Medically Assisted Reproduction in France Between 2010 and 2020 According to the French National Agency of Biomedicine (ABM) and in the Present Study

Supplement 2.
Data Sharing Statement
