# Supplementary material for: Medically Assisted Reproduction and Risk of Cancer Among Offspring
Source: JAMA Netw Open. 2024 May 2;7(5):e249429. doi: 10.1001/jamanetworkopen.2024.9429 (PMC11066701; doi:10.1001/jamanetworkopen.2024.9429)
Supplement: Supplement 1. — eTable 1. List of Codes Used for Identification of Medically Assisted Reproduction–Related Procedures eTable 2. Grouping of ICD-10 Diagnostic Codes Used to Approximate the International Childhood Cancer Classification eTable 3. Number of Cancers Among Children Born and Diagnosed in France Between 2010 and 2015 in the French National Registry of Childhood Cancers (RNCE) and in the Present Study eTable 4. Birth Characteristics of Children Born in France in 2010 to 2021 by Cancer Status eTable 5. Risk of Cancer in Children Born in France in 2010 to 2021 After Fresh Embryo Transfer Compared to Naturally Conceived Children eTable 6. Risk of Cancer in Children Born in France in 2010 to 2021 After Frozen Embryo Transfer Compared to Naturally Conceived Children eTable 7. Risk of Cancer in Children Born in France in 2010 to 2021 After Artificial Insemination Compared to Naturally Conceived Children eTable 8. Number of Cases, Incidence Rate, and Risk of Cancer in Children Born After Natural Conception, Fresh Embryo Transfer, Frozen Embryo Transfer, or Artificial Insemination in Singletons Born in France in 2010 to 2021 eTable 9. Risk of Cancer in Children Born in France in 2010 to 2021 After Fresh Embryo Transfer, Frozen Embryo Transfer, or Artificial Insemination Compared to Naturally Conceived Children eTable 10. Risk of Cancer in Children Born in France in 2010 to 2021 After Fresh Embryo Transfer, Frozen Embryo Transfer, or Artificial Insemination Compared to Naturally Conceived Children eTable 11. Number of Children Born After Medically Assisted Reproduction in France Between 2010 and 2020 According to the French National Agency of Biomedicine (ABM) and in the Present Study [file jamanetwopen-e249429-s001.pdf]

Supplementary Online Content

Rios P, Herlemont P, Fauque P, et al. Medically assisted reproduction and risk of cancer among offspring. *JAMA Netw Open*. 2024;7(5):e249429.
doi:10.1001/jamanetworkopen.2024.9429

eTable 1. List of Codes Used for Identification of Medically Assisted Reproduction–Related Procedures

eTable 2. Grouping of *ICD-10* Diagnostic Codes Used to Approximate the International Childhood Cancer Classification

eTable 3. Number of Cancers Among Children Born and Diagnosed in France Between 2010 and 2015 in the French National Registry of Childhood Cancers (RNCE) and in the Present Study

eTable 4. Birth Characteristics of Children Born in France in 2010 to 2021 by Cancer Status

eTable 5. Risk of Cancer in Children Born in France in 2010 to 2021 After Fresh Embryo Transfer Compared to Naturally Conceived Children

eTable 6. Risk of Cancer in Children Born in France in 2010 to 2021 After Frozen Embryo Transfer Compared to Naturally Conceived Children

eTable 7. Risk of Cancer in Children Born in France in 2010 to 2021 After Artificial Insemination Compared to Naturally Conceived Children

eTable 8. Number of Cases, Incidence Rate, and Risk of Cancer in Children Born After Natural Conception, Fresh Embryo Transfer, Frozen Embryo Transfer, or Artificial Insemination in Singletons Born in France in 2010 to 2021

eTable 9. Risk of Cancer in Children Born in France in 2010 to 2021 After Fresh Embryo Transfer, Frozen Embryo Transfer, or Artificial Insemination Compared to Naturally Conceived Children

eTable 10. Risk of Cancer in Children Born in France in 2010 to 2021 After Fresh Embryo Transfer, Frozen Embryo Transfer, or Artificial Insemination Compared to Naturally Conceived Children

eTable 11. Number of Children Born After Medically Assisted Reproduction in France Between 2010 and 2020 According to the French National Agency of Biomedicine (ABM) and in the Present Study

This supplementary material has been provided by the authors to give readers additional information about their work.

© 2024 Rios P et al. *JAMA Network Open.*

eTable 1. List of Codes Used for Identification of Medically Assisted Reproduction–Related Procedures

Common Classification of Medical Acts (CCAM) code	Label
JJFJ001 / JJFC011	Oocyte retrieval
JSED001 / JSEC001	Intra-uterine embryo transfer
JSLD001 / JSLD002	Artificial insemination

eTable 2. Grouping of *ICD-10* Diagnostic Codes Used to Approximate the International Childhood Cancer Classification

Diagnostic groups	ICD-10 codes
Leukaemias	C91-C95
Acute lymphoblastic leukaemia (ALL)	C91
Acute myeloid leukaemia (AML)	C92-C93
Lymphomas	C81-C85, C88, C90, C96
Malignant tumours or the central nervous system	C70-C72, C751, C752, C753
Tumours of adrenal glands, peripheral nerves and autonomic nervous system	C74.1, C74.9, C47, D482
Retinoblastomas	C69
Renal tumours	C64, C65
Hepatic tumours	C22
Malignant bone tumours	C40-C41
Soft tissue tumours	C01-C07, C13, C17, C21, C24, C26, C30, C31, C48, C49, C51, C52, C61, C63, C66, C67
Tumours of germ cells and gonads	C56, C57, C58, C62, C758, C75
Epithelial neoplasms and melanomas	C08, C10, C11, C12, C18, C19, C20, C23, C25, C32, C33, C34, C37, C43, C44, C50, C53, C54, C55, C60, C68, C73 C74.0
Other and unspecified malignant neoplasms	C14, C16, C38, C76

© 2024 Rios P et al. *JAMA Network Open.*

eTable 3. Number of Cancers Among Children Born and Diagnosed in France Between 2010 and 2015 in the French National Registry of Childhood Cancers (RNCE) and in the Present Study

	**RNCE** [a]	**Present study**	
	No.	No.	% of RNCE
Leukaemias	840	751	*89*
Acute lymphoblastic leukaemias	627	554	*88*
Acute myeloid leukaemias	173	180	*104*
Lymphomas	211	197	*93*
Malignant tumours or the central nervous system	415	535	*129*
Tumours of adrenal glands, peripheral nerves and autonomic nervous system	548	405	*74*
Retinoblastomas	230	231	*100*
Renal tumours	262	267	*102*
Hepatic tumours	72	80	*111*
Malignant bone tumours	16	50	*313*
Soft tissue sarcomas	184	182	*99*
Tumours of germ cells and gonads	95	62	*65*
Epithelial neoplasms and melanomas	38	105	*276*
Other and unspecified neoplasms	18	75	*417*
All cancers	2 929	2 879	*89*

[a] In the RNCE, the groups are those of the International classification of childhood cancer (ICCC-3) based on the morphology and topography codes of the International classification of diseases for oncology (ICDO-3), while in the present study grouping was approximated from the codes of the International classification of diseases (ICD10). Detailed information on the RNCE is available on https://rnce.inserm.fr/index.php/fr

© 2024 Rios P et al. *JAMA Network Open.*

eTable 4. Birth Characteristics of Children Born in France between 2010 and 2021 by Cancer Status

	Children without cancer [a]	Children with cancer [a]
	(n= 8 517 050)	(n= 9 256)
**Gestational length, weeks**		
≤28	25 316 (0.3)	26 (0.3)
29-32	58 319 (0.7)	72 (0.8)
33-36	515 495 (6.0)	625 (6.7)
37-41	7 846 527 (92.2)	8 452 (91.3)
≥ 42	71 393 (0.8)	81 (0.9)
**Foetal growth**		
Missing	258 398 (3.0)	345 (3.7)
Small for gestational age	1 024 589 (12.0)	991 (10.7)
Adequate for gestational age	6 330 126 (74.4)	6 644 (71.8)
Large for gestational age	903 937 (10.6)	1 276 (13.8)
**Congenital malformations**		
No	8 254 486 (96.9)	8 541 (92.3)
Yes	262 564 (3.1)	715 (7.7)
**Maternal age at index pregnancy, years**		
≤24	1 247 476 (14.6)	1 412 (15.2)
25-29	2 616 067 (30.7)	2 901 (31.3)
30-34	2 835 092 (33.4)	3 049 (32.9)
35-39	1 450 211 (17.0)	1 530 (16.7)
≥ 40	368 204 (4.3)	364 (3.9)
Maternal age, median (IQR), years	30 (17)	30 (5)
**Deprivation index**		
Missing	125 592 (1.5)	160 (1.7)
Overseas departments and regions	351 405 (4.1)	298 (3.2)
Q1 (least deprived)	1 624 292 (19.1)	1 752 (18.9)
Q2	1 622 829 (19.0)	1 800 (19.5)
Q3	1 589 612 (18.7)	1 744 (18.9)
Q4	1 548 419 (18.2)	1 660 (17.9)
Q5 (most deprived)	1 654 901 (19.4)	1 842 (19.9)

[a] data expressed as No. (%) unless otherwise indicated

© 2024 Rios P et al. *JAMA Network Open.*

eTable 5. Risk of Cancer in Children Born in France between 2010 and 2021 After Fresh Embryo Transfer Compared to Naturally Conceived Children. Statistical models adjusted for confounder factors and potential mediators.

	**Naturally conceived children**	**Children born after fresh embryo transfer**					
Childhood cancer types	Cases. No. (IR)	Cases. No. (IR)	HR (95% CI)[a]	HR (95% CI)[b]	HR (95% CI)[c]	HR (95% CI)[d]	HR (95% CI)[e]
Any cancer	8 964 (164)	165 (183)	1.11 (0.95-1.30)	1.12 (0.96-1.31)	1.13 (0.96-1.33)	1.13 (0.96-1.33)	1.13 (0.96-1.32)
Leukaemias	2 635 (48)	52 (58)	1.19 (0.90-1.56)	1.19 (0.90-1.56)	1.14 (0.85-1.51)	1.15 (0.85-1.51)	1.14 (0.84-1.50)
Acute lymphoblastic leukaemias	2 083 (38)	39 (43)	1.12 (0.82-1.54)	1.14 (0.83-1.57)	1.13 (0.81-1.57)	1.12 (0.81-1.57)	1.12 (0.80-1.57)
Acute myeloid leukaemias	475 (9)	10 (11)	1.28 (0.68-2.39)	1.20 (0.64-2.25)	1.10 (0.50-1.96)	1.10 (0.50-1.95)	1.03 (0.47-1.94)
Lymphomas	801 (15)	13 (14)	0.99 (0.57-1.69)	1.10 (0.63-1.90)	1.11 (0.65-1.98)	1.12 (0.65-1.98)	1.11 (0.65-1.97)
Malignant tumours of the central nervous system	1 813 (33)	35 (39)	1.16 (0.83-1.63)	1.23 (0.87-1.72)	1.29 (0.93-1.83)	1.29 (0.93-1.83)	1.29 (0.92-1.82)
Embryonal tumours	2 406 (44)	41 (45)	1.04 (0.76-1.41)	1.05 (0.77-1.43)	1.05 (0.76-1.44)	1.05 (0.76-1.44)	1.04 (0.75-1.43)
Tumours of adrenal glands[b]	941 (17)	20 (22)	1.29 (0.83-2.05)	1.35 (0.86-2.10)	1.34 (0.85-2.19)	1.34 (0.85-2.12)	1.33 (0.84-2.11)
Retinoblastomas	507 (9)	10 (11)	1.20 (0.64-2.25)	1.18 (0.63-2.23)	1.18 (0.68-2.42)	1.18 (0.68-2.42)	1.18 (0.68-2.41)
Renal tumours	777 (14)	10 (11)	0.79 (0.41-1.45)	0.81 (0.43-1.51)	0.79 (0.40-1.51)	0.78 (0.40-1.52)	0.78 (0.40-1.51)
Hepatic tumours	181 (3)	<5	-	-	-	-	-
Malignant bone tumours	235 (4)	<5	-	-	-	-	-
Soft tissue sarcomas	519 (10)	11 (12)	1.28 (0.71-2.33)	1.29 (0.70-2.35)	1.29 (0.71-2.36)	1.30 (0.75-2.54)	1.30 (0.75-2.53)
Tumours of germ cells and gonads	117 (2)	<5	-	-	-	-	-
Epithelial neoplasms and melanomas	306 (6)	8 (9)	1.58 (0.78-3.19)	1.32 (0.65-2.68)	1.39 (0.71-2.65)	1.39 (0.71-2.60)	1.39 (0.71-2.62)
Other and unspecified neoplasms	132 (2)	<5	-	-	-	-	-

[a] Crude hazard ratios and 95% CI

[b] Hazard ratios and 95% confidence intervals estimated by Cox models adjusted for year of birth, sex, multiple birth, maternal age and deprivation index

[c] Hazard ratios and 95% confidence intervals estimated by Cox models adjusted for year of birth, sex, multiple birth, maternal, deprivation index, gestational age and birth weight

[d] Hazard ratios and 95% confidence intervals estimated by Cox models adjusted for year of birth, sex, multiple birth, maternal, deprivation index, gestational age and fœtal macrosomia (LGA or > 4000 g)

[e] Hazard ratios and 95% confidence intervals estimated by Cox models adjusted for year of birth, sex, multiple birth, maternal, deprivation index, gestational age and congenital malformations

© 2024 Rios P et al. *JAMA Network Open.*

eTable 6. Risk of Cancer in Children Born in France between 2010 and 2021 After Frozen Embryo Transfer Compared to Naturally Conceived Children. Statistical models adjusted for confounder factors and potential mediators.

	**Naturally conceived children**	**Children born after frozen embryo transfer**					
Childhood cancer types	Cases. No. (IR)	Cases. No. (IR)	HR (95% CI)[a]	HR (95% CI)[b]	HR (95% CI)[c]	HR (95% CI)[d]	HR (95% CI)[e]
Any cancer	8 964 (164)	57 (172)	0.97 (0.75-1.26)	1.02 (0.78-1.32)	1.00 (0.77-1.31)	1.01 (0.77-1.31)	0.99 (0.76-1.30)
Leukaemias	2 635 (48)	23 (69)	1.37 (0.91-2.07)	1.42 (0.94-2.14)	1.45 (0.96-2.21)	1.46 (0.97-2.21)	1.45 (0.96-2.19)
Acute lymphoblastic leukaemias	2 083 (38)	20 (60)	1.54 (0.99-2.39)	1.61 (1.04-2.50)	1.66 (1.06-2.59)	1.66 (1.06-2.59)	1.66 (1.07-2.58)
Acute myeloid leukaemias	475 (9)	<5	-	-	-	-	-
Lymphomas	801 (15)	<5	-	-	-	-	-
Malignant tumours of the central nervous system	1 813 (33)	8 (24)	0.70 (0.35-1.41)	0.78 (0.39-1.59)	0.76 (0.34-1.49)	0.77 (0.34-1.48)	0.72 (0.34-1.48)
Embryonal tumours	2 406 (44)	16 (48)	0.93 (0.57-1.52)	0.96 (0.59-1.57)	0.93 (0.55-1.52)	0.92 (0.55-1.52)	0.91 (0.54-1.51)
Tumours of adrenal glands[b]	941 (17)	6 (18)	0.88 (0.39-1.96)	0.94 (0.42-2.09)	0.80 (0.33-1.93)	0.80 (0.33-1.93)	0.79 (0.34-1.91)
Retinoblastomas	507 (9)	<5	-	-	-	-	-
Renal tumours	777 (14)	7 (21)	1.31 (0.63-2.77)	1.36 (0.64-2.85)	1.37 (0.65-2.88)	1.37 (0.65-2.88)	1.35 (0.64-2.84)
Hepatic tumours	181 (3)	0	-	-	-	-	-
Malignant bone tumours	235 (4)	0	-	-	-	-	-
Soft tissue sarcomas	519 (10)	<5	-	-	-	-	-
Tumours of germ cells and gonads	117 (2)	<5	-	-	-	-	-
Epithelial neoplasms and melanomas	306 (6)	<5	-	-	-	-	-
Other and unspecified neoplasms	132 (2)	0	-	-	-	-	-

[a] Crude hazard ratios and 95% CI

[b] Hazard ratios and 95% confidence intervals estimated by Cox models adjusted for year of birth, sex, multiple birth, maternal age and deprivation index

[c] Hazard ratios and 95% confidence intervals estimated by Cox models adjusted for year of birth, sex, multiple birth, maternal, deprivation index, gestational age and birth weight

[d] Hazard ratios and 95% confidence intervals estimated by Cox models adjusted for year of birth, sex, multiple birth, maternal, deprivation index, gestational age and fœtal macrosomia (LGA or > 4000 g)

[e] Hazard ratios and 95% confidence intervals estimated by Cox models adjusted for year of birth, sex, multiple birth, maternal, deprivation index, gestational age and congenital malformations

© 2024 Rios P et al. *JAMA Network Open.*

eTable 7. Risk of Cancer in Children Born in France between 2010 and 2021 After Artificial Insemination Compared to Naturally Conceived Children .Statistical models adjusted for confounder factors and potential mediators.

	**Naturally conceived children**	**Children born after artificial insemination**					
Childhood cancer types	Cases, No. (IR)	Cases, No. (IR)	HR (95% CI)[a]	HR (95% CI)[b]	HR (95% CI)[c]	HR (95% CI)[d]	HR (95% CI)[e]
Any cancer	8 964 (164)	70 (179)	1.08 (0.89 - 1.37)	1.09 (0.86-1.38)	1.08 (1.85 - 1.38)	1.08 (1.85 - 1.38)	1.08 (1.85 - 1.38)
Leukaemias	2 635 (48)	19 (49)	1.00 (0.64-1.57)	1.01 (0.64-1.58)	1.00 (0.63-1.59)	1.00 (0.63-1.59)	1.00 (0.63-1.59)
Acute lymphoblastic leukaemias	2 083 (38)	16 (41)	1.07 (0.65-1.74)	1.09 (0.66-1.78)	1.09 (0.65-1.80)	1.09 (0.65-1.80)	1.08 (0.65-1.80)
Acute myeloid leukaemias	475 (9)	<5	-	-	-	-	-
Lymphomas	801 (15)	6 (15)	1.05 (0.47-2.34)	1.13 (0.50-2.52)	1.10 (0.29-2.09)	1.10 (0.29-2.09)	1.10 (0.29-2.09)
Malignant tumours of the central nervous system	1 813 (33)	19 (48)	1.46 (0.93-2.29)	1.51 (0.92-2.38)	1.53 (0.95-2.43)	1.53 (0.95-2.43)	1.53 (0.96-2.43)
Embryonal tumours	2 406 (44)	18 (46)	1.03 (0.65-1.64)	1.05 (0.66-1.67)	1.10 (0.69-1.75)	1.10 (0.69-1.75)	1.10 (0.69-1.75)
Tumours of adrenal glands[b]	941 (17)	9 (23)	1.32 (0.68-2.54)	1.38 (0.72-2.77)	1.40 (0.75-2.79)	1.40 (0.75-2.79)	1.40 (0.75-2.79)
Retinoblastomas	507 (9)	<5	-	-	-	-	-
Renal tumours	777 (14)	6 (15)	1.07 (0.45-2.39)	1.09 (0.49-2.45)	1.16 (0.52-2.60)	1.16 (0.52-2.60)	1.16 (0.52-2.60)
Hepatic tumours	181 (3)	<5	-	-	-	-	-
Malignant bone tumours	235 (4)	<5	-	-	-	-	-
Soft tissue sarcomas	519 (10)	<5	-	-	-	-	-
Tumours of germ cells and gonads	117 (2)	0	-	-	-	-	-
Epithelial neoplasms and melanomas	306 (6)	<5	-	-	-	-	-
Other and unspecified neoplasms	132 (2)	<5	-	-	-	-	-

[a] Crude hazard ratios and 95% CI

[b] Hazard ratios and 95% confidence intervals estimated by Cox models adjusted for year of birth, sex, multiple birth, maternal age and deprivation index

[c] Hazard ratios and 95% confidence intervals estimated by Cox models adjusted for year of birth, sex, multiple birth, maternal, deprivation index, gestational age and birth weight

[d] Hazard ratios and 95% confidence intervals estimated by Cox models adjusted for year of birth, sex, multiple birth, maternal, deprivation index, gestational age and fœtal macrosomia (LGA or > 4000 g)

[e] Hazard ratios and 95% confidence intervals estimated by Cox models adjusted for year of birth, sex, multiple birth, maternal, deprivation index, gestational age and congenital malformations

© 2024 Rios P et al. *JAMA Network Open.*

© 2024 Rios P et al. *JAMA Network Open.*

eTable 8. Number of Cases, Incidence Rate, and Risk of Cancer in Children Born After Natural Conception, Fresh Embryo Transfer, Frozen Embryo Transfer, or Artificial Insemination in Singletons Born in France between 2010 and 2021

		**Children born after medically assisted reproduction**					
	**Naturally conceived children**	**Children born after fresh embryo transfer**		**Children born after frozen embryo transfer**		**Children born after artificial insemination**	
	No. (PY) 8 013 531 (52 869 242)	No. (PY) 101 862 (664 650)		No. (PY) 56 177 (273 759)		No. (PY) 48 364 (312 112)	
**Childhood cancer type**	Cases, No. (IR)	Cases, No. (IR)	HR (95% CI)[a]	Cases, No. (IR)	Cases, No. (IR)	Cases, No. (IR)	HR (95% CI)[a]
Any type of childhood cancer	8 701 (165)	120 (181)	1.09 (0.91-1.31)	50 (183)	1.07 (0.81-1.41)	58 (185)	1.12 (0.87-1.45)
Leukaemias	2 558 (48)	33 (50)	1.02 (0.73-1.44)	21 (77)	1.59 (1.01-2.39)	15 (48)	0.99 (0.59-1.64)
Acute lymphoblastic leukaemias	2 028 (38)	24 (36)	0.94 (0.63_1.40)	18 (65)	1.73 (1.09-2.75)	14 (44)	1.16 (0.68-1.97)
Acute myeloid leukaemias	456 (9)	6 (9)	1.04 (0.46-2.32)	<5	-	<5	-
Lymphomas	787 (15)	12 (18)	1.23 (0.69-2.17)	<5	-	6 (19)	1.30 (0.58-2.90)
Malignant tumours of the central nervous system	1 761 (33)	27 (40)	1.25 (0.86-1.84)	8 (29)	0.92 (0.46-1.84)	14 (45)	1.37 (0.81-2.32)
Tumours of adrenal glands [b]	921 (17)	12 (18)	1.03 (0.58-1.81)	<5	-	8 (25)	1.45 (0.73-2.91)
Retinoblastomas	489 (9)	6 (9)	0.98 (0.45-2.20)	<5	-	<5	-
Renal tumours	757 (14)	9 (13)	0.93 (0.45-1.80)	6 (22)	1.36 (0.61-3.04)	5	1.10 (0.46-2.66)
Hepatic tumours	171 (3)	<5	-	<5	-	<5	-
Malignant bone tumours	224 (4)	<5	-	<5	-	<5	-
Soft tissue sarcomas	502 (9)	11 (17)	1.73 (0.96-3.15)	<5	-	<5	-
Tumours of germ cells and gonads	113 (2)	<5	-	<5	-	<5	-
Epithelial neoplasms and melanomas	290 (6)	6	1.65 (0.74-3.71)	<5	-	<5	-
Other and unspecified neoplasms	128 (2)	<5	-	<5	-	<5	-

Abbreviations: PY: No. of person-years; IR: Incidence rate/million person-years; HR: hazard ratio; CI: confidence interval;

[a] Adjusted for year of birth, sex, multiple birth, maternal age and deprivation index

[b] Includes tumours of adrenal glands, peripheral nerves and autonomic nervous system

Estimates are not provided when less than 5 exposed cases

© 2024 Rios P et al. *JAMA Network Open.*

eTable 9. Risk of Cancer in Children Born in France between 2010 and 2021 After Fresh Embryo Transfer, Frozen Embryo Transfer, or Artificial Insemination Compared to Naturally Conceived Children. First cancer diagnosis considered for cancer type classification

		**Children born after medically assisted reproduction**					
	**Naturally conceived children**	**Children born after fresh embryo transfer**		**Children born after frozen embryo transfer**		**Children born after artificial insemination**	
Childhood cancer types	Cases, No. (IR)	Cases, No. (IR)	HR (95% CI)[a]	Cases, No. (IR)	HR (95% CI)[a]	Cases, No. (IR)	HR (95% CI)[a]
Any cancer	8 964 (164)	165 (183)	1.12 (0.96-1.31)	57 (172)	1.02 (0.78-1.32)	70 (179)	1.09 (0.86-1.38)
Leukaemias	2627 (48)	52 (58)	1.19 (0.90-1.56)	23 (69)	1.42 (0.94-2.14)	19 (49)	1.01 (0.64-1.58)
Acute lymphoblastic leukaemias	1 904 (35)	37 (41)	1.18 (0.85-1.65)	19 (57)	1.67 (1.06-2.63)	16 (41)	1.09 (0.66-1.78)
Acute myeloid leukaemias	430 (8)	7 (8)	0.93 (0.44-1.97)	<5	-	<5	-
Lymphomas	749 (14)	13 (14)	1.10 (0.63-1.90)	<5	-	5 (13)	1.13 (0.50-2.52)
Malignant tumours of the central nervous system	1836 (34)	35 (39)	1.23 (0.87-1.72)	9 (27)	0.86 (0.45-1.66)	20 (51)	1.58 (1.00 -2.45)
Embryonal tumours	2 376 (44)	43 (47)	1.12 (0.83-1.52)	15 (45)	0.92 (0.55-1.53)	18 (46)	1.05 (0.66-1.67)
Tumours of adrenal glands[b]	847 (15)	20 (22)	1.35 (0.86-2.10)	<5	-	9 (23)	1.38 (0.72-2.77)
Retinoblastomas	518 (9)	10 (11)	1.18 (0.63-2.23)	<5	-	<5	-
Renal tumours	821 (15)	12 (13)	0.91 (0.51-1.62)	7 (21)	1.36 (0.64-2.85)	6 (15)	1.09 (0.49-2.45)
Hepatic tumours	190 (3)	<5	-	<5	-	<5	-
Malignant bone tumours	238 (4)	<5	-	0	-	<5	-
Soft tissue sarcomas	352 (6)	6 (7)	1.05 (0.47-2.38)	<5	-	<5	-
Tumours of germ cells and gonads	150 (3)	<5	-	<5	-	<5	-
Epithelial neoplasms and melanomas	75 (2)	<5	-	0	-	<5	-
Other and unspecified neoplasms	561 (10)	10 (11)		<5	-	<5	-

Abbreviations: PY: No. of person-years; IR: Incidence rate/million person-years; HR: hazard ratio; CI: confidence interval;

[a] Hazard ratios and confidence intervals estimated by Cox regression models adjusted for year of birth, sex, multiple birth, maternal age and deprivation index

[b] Includes tumours of adrenal glands, peripheral nerves and autonomic nervous system;

Estimates are not provided when less than 5 exposed cases

© 2024 Rios P et al. *JAMA Network Open.*

eTable 10. Risk of Cancer in Children Born in France between 2010 and 2021 After Fresh Embryo Transfer, Frozen Embryo Transfer, or Artificial Insemination Compared to Naturally Conceived Children. Cases with chemotherapy exposure only.

		Children born after medically assisted reproduction					
	**Naturally conceived children**	**Children born after fresh embryo transfer**		**Children born after frozen embryo transfer**		**Children born after artificial insemination**	
Childhood cancer types	Cases, No. (IR)	Cases, No. (IR)	HR (95% CI)[a]	Cases, No. (IR)	HR (95% CI)[a]	Cases, No. (IR)	HR (95% CI)[a]
Any cancer	7 100 (130)	131 (145)	1.14 (0.96-1.36)	47 (141)	1.07 (0.80-1.42)	58 (148)	1.15 (0.89-1.50)
Leukaemias	2 547 (47)	52 (58)	1.24 (0.94-1.64)	23 (69)	1.49 (0.99-2.25)	18 (46)	0.99 (0.62-1.58)
Acute lymphoblastic leukaemias	2 059 (38)	39 (43)	1.16 (0.84-1.60)	20 (60)	1.64 (1.05-2.55)	16 (41)	1.10 (0.67-1.80)
Acute myeloid leukaemias	425 (8)	10 (11)	1.37 (0.73-2.58)	<5	-	<5	-
Lymphomas	656 (12)	10 (11)	1.04 (0.56-1.96)	<5	-	5 (13)	1.16 (0.48-2.81)
Malignant tumours of the central nervous system	1 160 (21)	23 (25)	1.29 (0.85-1.95)	6 (18)	0.88 (0.39-1.96)	13 (33)	1.59 (0.92-2.77)
Embryonal tumours	1 919 (35)	32 (35)	1.03 (0.73-1.47)	13 (39)	0.99 (0.58-1.72)	16 (41)	1.18 (0.72-1.93)
Tumours of adrenal glands[b]	693 (12)	13 (14)	1.21 (0.69-2.11)	5 (15)	1.09 (0.45-2.63)	8 (20)	1.69 (0.84-3.41)
Retinoblastomas	338 (6)	9 (10)	1.58 (0.81-3.09)	<5	-	<5	-
Renal tumours	729 (13)	9 (10)	0.78 (0.40-1.51)	6 (18)	1.25 (0.56-2.80)	6 (15)	1.18 (0.52-2.63)
Hepatic tumours	159 (3)	<5	-	0		<5	-
Malignant bone tumours	191 (4)	<5	-	0		0	-
Soft tissue sarcomas	397 (7)	9 (10)		<5		0	-
Tumours of germ cells and gonads	44 (1)	<5	-	0		<5	-
Epithelial neoplasms and melanomas	121 (2)	<5	-	<5		0	-
Other and unspecified neoplasms	65 (1)	0	-	0		<5	-

Abbreviations: PY: No. of person-years; IR: Incidence rate/million person-years; HR: hazard ratio; CI: confidence interval;

[a] Hazard ratios and confidence intervals estimated by Cox regression models adjusted for year of birth, sex, multiple birth, maternal age and deprivation index

[b] Includes tumours of adrenal glands, peripheral nerves and autonomic nervous system;

Estimates are not provided when less than 5 exposed cases

© 2024 Rios P et al. *JAMA Network Open.*

eTable 11. Number of Children Born After Medically Assisted Reproduction in France Between 2010 and 2020 According to the French National Agency of Biomedicine (ABM) and in the Present Study

	**ABM [a]**	**Present study**	
	No.	No.	% of ABM
Fresh embryo transfer	134 522	125 769	93%
Frozen embryo transfer	64 420	57 376	89%
Artificial insemination	66 800	55 554	83%
Total	265 742	238 699	90%

[a] Annual reports available until 2020 on https://www.agence-biomedecine.fr/Site-des-professionnels.

© 2024 Rios P et al. *JAMA Network Open.*
