# Supplementary material for: Medically Assisted Reproduction and Risk of Cancer Among Offspring
Source: JAMA Netw Open. 2024 May 2;7(5):e249429. doi: 10.1001/jamanetworkopen.2024.9429 (PMC11066701; doi:10.1001/jamanetworkopen.2024.9429)
Supplement: Supplement 2. — Data Sharing Statement [file jamanetwopen-e249429-s002.pdf]

Data Sharing Statement

Rios. Medically Assisted Reproduction and Risk of Cancer Among Offspring. *JAMA Netw Open*. Published May 02, 2024. doi:10.1001/jamanetworkopen.2024.9429

Data
Data available: No
